# Impact of COVID-19 on pediatric surgical practice in Taiwan: a comprehensive analysis

**DOI:** 10.3389/fped.2024.1354576

**Published:** 2024-04-17

**Authors:** Sheng-Yang Huang, Chia-Man Chou, Hou-Chuan Chen

**Affiliations:** ^1^ Division of Pediatric Surgery, Department of Surgery, Taichung Veterans General Hospital, Taichung, Taiwan; ^2^ School of Medicine, College of Medicine, National Yang Ming Chiao Tung University, Taipei, Taiwan; ^3^ Department of Post-Baccalaureate Medicine, College of Medicine, National Chung Hsing University, Taichung, Taiwan

**Keywords:** COVID-19, pediatric surgery, healthcare disruption, Taiwan, surgical practice, public health emergency

## Abstract

**Background:**

The COVID-19 pandemic has profoundly impacted global healthcare systems, causing significant disruptions in various medical practices. This study focuses on the specific effects of the pandemic on pediatric surgical practice in Taiwan, a region known for its effective public health measures and proximity to the initial outbreak.

**Methods:**

The study analyzes data from January 2020 to August 2022, comparing it with historical records from January 2017 to August 2019. It examines changes in surgical case volumes, patient demographics, surgical indications, and trends in preoperative evaluations, surgical procedures, and postoperative care.

**Results:**

The study reveals a decrease in total surgical cases from 2,255 to 1,931 during the pandemic. Notable findings include a slight increase in the average age of patients (4.81 to 5.10 years, *p* = 0.064), a significant shift in gender distribution towards male patients (68.9% to 73.5%, *p* = 0.0009), and changes in the types of surgical procedures performed, with head and neck and gastrointestinal surgeries seeing an increase. The average hospital stay lengthened, and certain specific surgical diseases, like hypospadias and liver tumors, showed an increase. However, the age distribution of pediatric surgical patients remained stable, and emergency surgical care was resiliently maintained.

**Discussion:**

The findings demonstrate the adaptability of Taiwan's healthcare system in maintaining pediatric surgical care during the pandemic. The study highlights a significant gender disparity in surgical interventions and a shift towards more urgent and emergent care, reflecting the reorganization of healthcare services during this period. The study's limitations include its retrospective nature and focus on a single institution.

**Conclusion:**

This research contributes valuable insights into the impact of the COVID-19 pandemic on pediatric surgical practice in Taiwan. It underscores the importance of adaptable healthcare strategies in ensuring continuity and quality of care during public health emergencies. Future research should focus on multi-institutional data and prospective studies to further understand these dynamics.

## 1 Introduction

The Coronavirus (COVID-19) pandemic, which emerged in late 2019, rapidly escalated into a global health crisis, challenging healthcare systems and causing widespread disruptions (1). As the virus spread across borders, healthcare professionals and researchers worldwide grappled with its multifaceted impact on medical practices, patient care, and healthcare infrastructure (2). One such area significantly affected was surgical practice, where elective procedures were postponed, and healthcare systems faced unprecedented challenges in maintaining patient safety amidst the pandemic (3–7).

Taiwan, situated in the proximity of the initial outbreak, was among the first countries to face the challenges posed by COVID-19. The nation, known for its proactive approach to public health emergencies, swiftly implemented stringent measures to curb the spread of the virus. Through a combination of early identification, contact tracing, and strict quarantine protocols, Taiwan managed to keep COVID-19 cases relatively low and contained, effectively preventing overwhelming pressure on its healthcare system (2, 8, 9).

Despite the successful containment efforts, the healthcare landscape in Taiwan, as in other regions, faced significant disruptions due to the COVID-19 pandemic. The reallocation of hospital resources and the adjustment of protocols were essential to maintain patient and healthcare worker safety during this period (10). Pediatric surgical practice, in particular, presented unique challenges due to the specialized care required for this vulnerable population, ranging from congenital anomalies to urgent surgical emergencies (11, 12).

This research aims to elucidate the nuanced impact of the COVID-19 pandemic on pediatric surgical practice in Taiwan. By examining data from multiple years, encompassing both pandemic and non-pandemic periods, we strive to identify trends affected by the pandemic. Primary objectives include elucidating the pandemic's effect on the volume and nature of surgical cases and identifying demographic shifts within the patient population. Secondary objectives involve analyzing changes in surgical indications. Such an analysis is pivotal in understanding the adjustments necessitated by the pandemic, including changes in preoperative evaluations, surgical procedures, and postoperative care (13). The insights gained from this study are intended to contribute to the broader discourse on healthcare resilience and inform future preparedness efforts within the medical community (14).

## 2 Materials and methods

The study is a retrospective cohort study, which investigated the periods of COVID-19 outbreaks and relatively stable phases from January 2020 to August 2022. It examined the volume and characteristics of pediatric surgical patients treated at the institution during these different periods, comparing them with historical records from non-pandemic years (January 2017 to August 2019). For the given time period, all patients who underwent surgical interventions in our department were continuously enrolled without any exclusions. Informed consent was exempted according to the instructions of Institutional Review Board I of our institute (certificate No. CE22382A). This analysis aimed to identify changes in the number of surgical cases and disease categories attributed to the COVID-19 pandemic. Data collection included surgical case volumes, patient demographics, surgical indications, and outcomes. All de-identified data were stored on a separate server, securely locked in the corresponding author's office and subject to access control. Data analysis was performed using MedCalc® Statistical Software version 22.013, accessed on 6th October, 2023 (MedCalc Software Ltd., Ostend, Belgium; https://www.medcalc.org; 2023). Continuous variables were expressed as mean values (95% confidential interval), and categorical variables were expressed as a number of patients (percentage). Comparisons between groups were performed using the *t*-test for continuous variables and the chi-squared test for categorical variables. The significance was set as *p*-value < 0.05.

## 3 Results

### 3.1 Socio-demographics

As shown in Table 1, The data indicated that the mean age of surgical patients during these two periods did not exhibit a statistically significant difference. While a slight difference in mean age was observed, the *p*-value (0.064) exceeded the significance threshold, suggesting that the age distribution of pediatric surgical patients did not show substantial variance between the pandemic and non-pandemic periods.

**Table 1:** Patient characteristics of non-pandemic and pandemic periods.

Group	Non-pandemic (2017–2019), *n* = 2,255	Pandemic (2020–2022), *n* = 1,931	*p* value
Socio-demographics			
Age, year	4.81 (4.61 to 5.02)	5.10 (4.87 to 5.33)	0.064
Male gender	1,554 (68.9%)	1,421 (73.5%)	0.0009
Categories of surgery			
Regular	2,088 (92.6%)	1,777 (92.0%)	0.490
Emergent	167 (7.4%)	154 (8.0%)	
Day-care	656 (29.0%)	532 (27.5%)	0.270
Surgical sites			
Head and neck	81 (3.6%)	102 (5.3%)	0.007
Chest	143 (6.3%)	124 (6.4%)	0.915
Gastrointestinal system	206 (9.1%)	227 (11.7%)	0.005
Genitourinary system	340 (15.1%)	330 (17.1%)	0.076
Colorectum	139 (6.2%)	111 (5.7%)	0.571
Inguinal hernia	570 (25.3%)	435 (22.5%)	0.037
Undescended testis	169 (7.5%)	120 (6.2%)	0.103
Excision	297 (13.2%)	243 (12.6%)	0.572
Others	310 (13.7%)	239 (12.4%)	0.190
Outcomes			
Hospital stay, day	6.35 (5.23 to 7.46)	6.80 (6.20 to 7.40)	0.001
Wound infection	5 (0.2%)	5 (0.3%)	0.805
Complication	9 (0.4%)	6 (0.3%)	0.633
Surgical mortality	0	1 (0.05%)	0.279

In the pandemic period, more male pediatric patients underwent surgical intervention. The *p*-value (0.0009) was below the significance threshold, indicating that the observed variance in the gender distribution was unlikely to be a result of random chance.

### 3.2 Differences in surgical cases

Patient characteristics of non-pandemic and pandemic periods are listed in Table 1. Total surgical cases decreased from 2,255 to 1,931. During the pandemic, there was an increase in the average age of patients, rising from 4.81 years to 5.10 years, although this change was not deemed significant (*p* = 0.064). A significant shift was observed in the gender distribution, with male patients increasing from 68.9% to 73.5% during the pandemic (*p* = 0.0009). Regular cases decreased slightly, while emergent cases increased, but these changes were not statistically significant. Day-care patients decreased marginally during the pandemic, from 29.0% to 27.5%. A significant increase in the average hospital stay was noted, from 6.35 days to 6.80 days (*p* = 0.001). Surgical procedures also saw significant shifts; head and neck surgeries increased from 3.6% to 5.3% (*p* = 0.007), and gastrointestinal system surgeries rose from 9.1% to 11.7% (*p* = 0.005), while inguinal hernia surgeries decreased (*p* = 0.037). Other surgical locations, along with wound infections, complications, and surgical mortality, did not show significant changes.

For specific surgical diseases, called index surgery, the patient numbers of non-pandemic and pandemic periods are listed in Table 2. Conditions such as congenital diaphragmatic hernia, biliary atresia, abdominal wall defects, anorectal malformations, hypospadias, and liver tumors increased during the pandemic period. Other conditions decreased or remained stable. All the changes were not significantly different.

**Table 2:** Patient number for index surgery of non-pandemic and pandemic periods.

Group	Non-pandemic (2017–2019), *n* = 2,255	Pandemic (2020–2022), *n* = 1,931	*p* value
Esophageal atresia	5 (0.22%)	2 (0.10%)	0.351
Congenital diaphragmatic hernia	6 (0.27%)	6 (0.31%)	0.788
Choledochal cyst	13 (0.58%)	10 (0.52%)	0.798
Biliary atresia, Kasai's operation	3 (0.13%)	6 (0.31%)	0.216
Abdominal wall defect	2 (0.09%)	3 (0.16%)	0.534
Duodenal & intestinal atresia	8 (0.35%)	4 (0.21%)	0.373
Extensive necrotizing enterocolitis	12 (0.53%)	10 (0.52%)	0.949
Anorectal malformation	17 (0.75%)	22 (1.14%)	0.196
Hirschsprung's disease	7 (0.31%)	6 (0.31%)	0.999
Ureteropelvic junction obstruction	14 (0.62%)	11 (0.57%)	0.830
Vesicoureteral reflux	9 (0.40%)	11 (0.57%)	0.425
Hypospadias	48 (2.13%)	58 (3.00%)	0.072
Liver tumor	0 (0.00%)	3 (0.16%)	0.061
Total	144 (6.39%)	152 (7.87%)	0.062

### 3.3 Categories of surgery

Regarding emergent operations, as shown in Table 1, the results indicated that there was no statistically significant difference in the proportion of emergency surgeries during the COVID-19 and non-COVID-19 periods.

**Figure 1:**
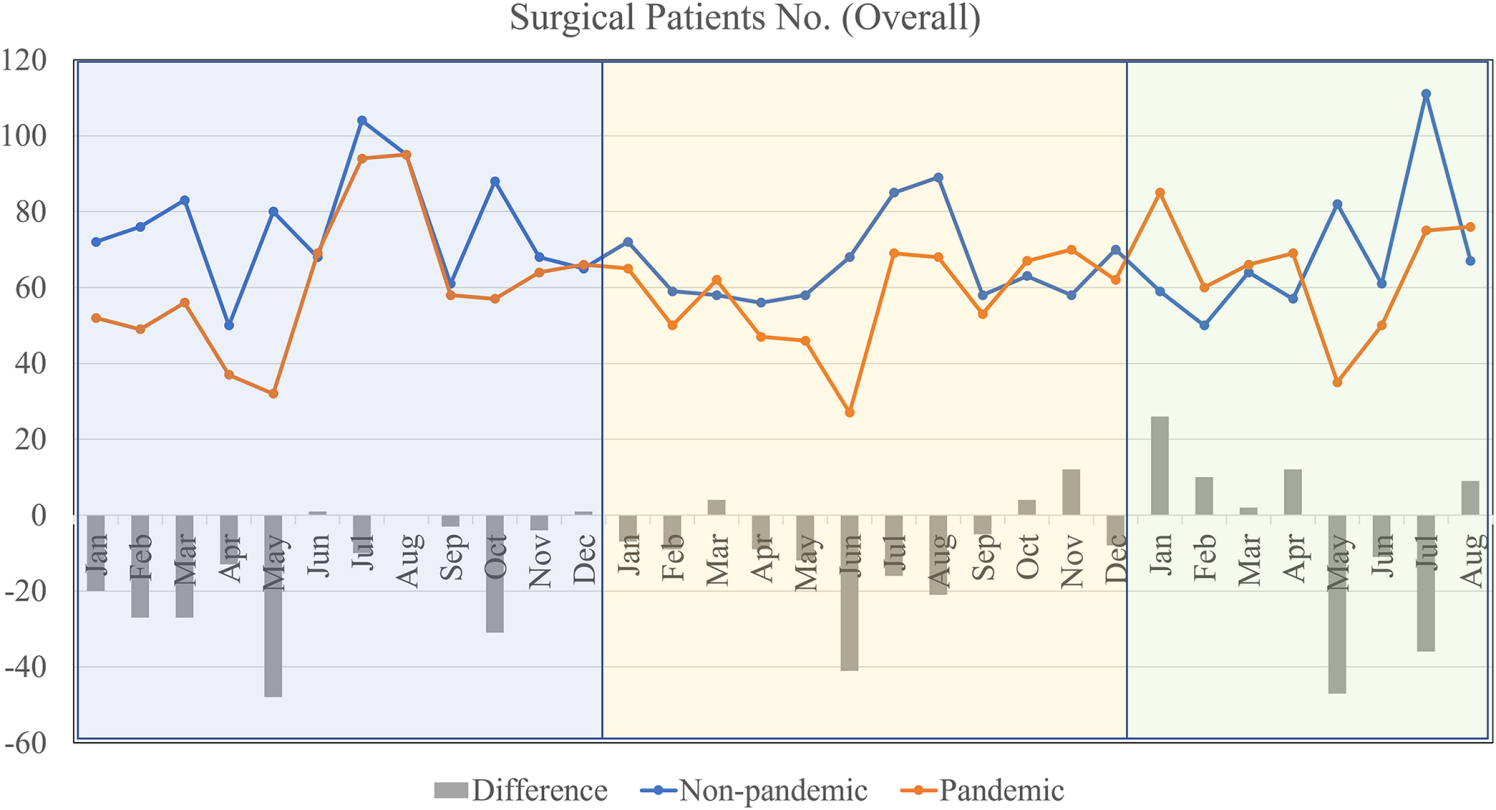
Total surgeries by months during non-pandemic and pandemic periods.

In Figure 1, it provides a broader view of the total number of surgical patients during the pandemic and non-pandemic periods. Both non-pandemic and pandemic lines show fluctuations throughout the year. The “Difference” bar indicates that during some months, notably April, June, and August, there was a significant decrease in the number of surgeries performed during the pandemic compared to non-pandemic periods. Conversely, months like January and December show a lesser difference in numbers.

**Figure 2:**
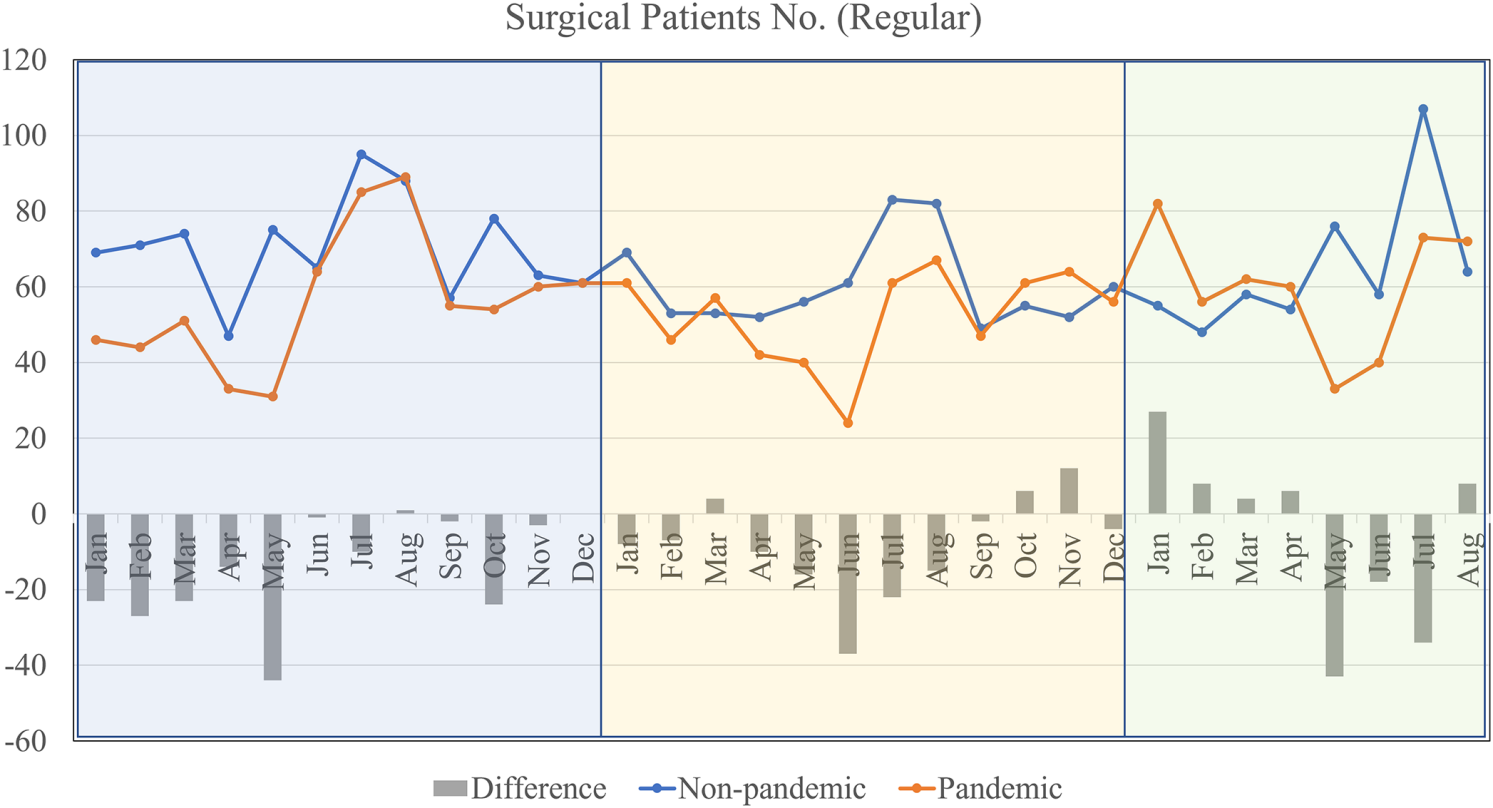
Regular surgeries by months during non-pandemic and pandemic periods.

The number of regular or non-emergency surgical cases was depicted in Figure 2. The non-pandemic line consistently remains above the pandemic line, indicating that regular surgeries were frequently postponed or canceled during the pandemic. The most significant decreases during the pandemic, as indicated by the “Difference” bars, occur in months like April, June, and August. There's a sharp rise in regular surgeries after June in both lines, possibly due to the resumption of previously postponed surgeries.

**Figure 3:**
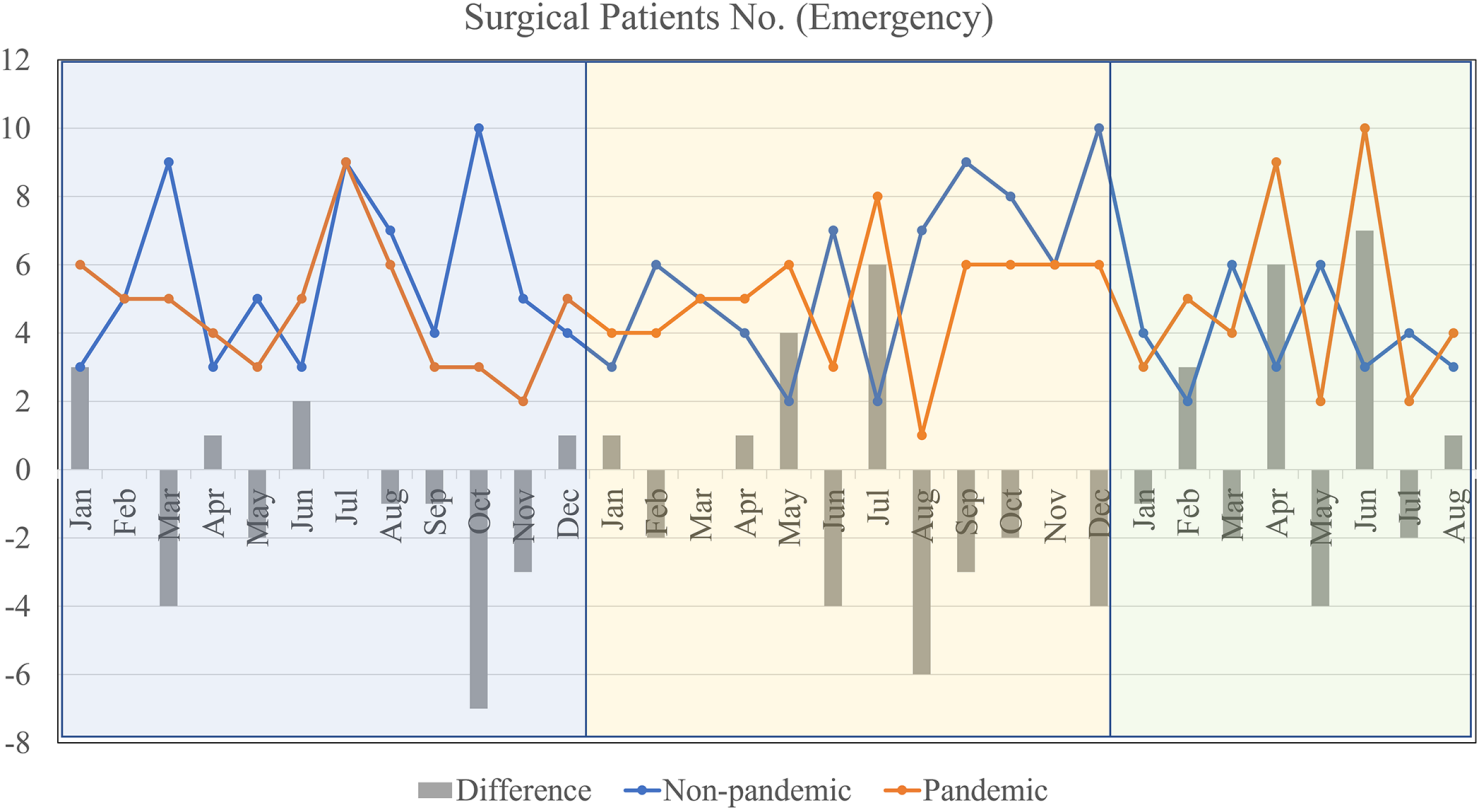
Emergent surgeries by months during non-pandemic and pandemic periods.

For emergent cases by months, the graphic illustration was as Figure 3. Both pandemic and non-pandemic lines show a degree of similarity in terms of fluctuations, indicating that emergency cases were not drastically affected by the pandemic. However, there are still notable drops in the number of emergency surgeries during the pandemic in months like January, April, and June. It's interesting to note that despite the pandemic, emergency surgeries have sometimes exceeded or matched non-pandemic numbers in months like February and July.

## 4 Discussion

The COVID-19 pandemic, a global health crisis of unprecedented scale, has profoundly impacted various aspects of healthcare systems across the globe. In Taiwan, renowned for its efficient and effective containment measures, the pandemic has nonetheless exerted significant disruptions, particularly in the realm of pediatric surgical practice. The focus of this study is to delve into the nuanced ways in which these disruptions have manifested. By conducting a comprehensive analysis of surgical case volumes, patient demographics, and indications for surgery during the pandemic, the study aims to elucidate the changes and challenges faced by pediatric surgeons and healthcare facilities. This entails a comparative approach, juxtaposing pandemic-era data against pre-pandemic figures to identify trends, shifts in surgical priorities, and alterations in patient care strategies. Furthermore, the study investigates the operational adjustments and innovations adopted by healthcare providers to navigate the constraints imposed by the pandemic, such as the increased reliance on telemedicine and adjustments in triage protocols. Through this exploration, the research seeks to offer insights into the resilience and adaptability of the healthcare sector, particularly in pediatric surgery, in the face of an unparalleled global health emergency.

### 4.1 Socio-demographics

The analysis of pediatric surgical patients' ages during the COVID-19 pandemic, compared to a non-pandemic period, presents intriguing findings that contribute to the broader understanding of how healthcare systems have navigated the challenges posed by the pandemic. Despite the significant upheavals in healthcare delivery and the expected shifts in patient demographics due to the pandemic's impact, the age distribution among pediatric surgical patients remained remarkably stable (4.81 years vs. 5.10 years, respectively). Though the trend of aged surgical patients during pandemics was observed. This stability suggests an underlying resilience in the healthcare system's ability to continue providing essential surgical services to pediatric patients, irrespective of their age. Such consistency is noteworthy, especially considering the extensive reorganization of healthcare services necessitated by the pandemic (15). This reorganization included the reallocation of healthcare resources, adjustments in healthcare provision priorities, and the introduction of stringent infection control measures. The fact that the demographic characteristics of pediatric surgical patients have not shifted significantly reinforces the notion that pediatric surgical care has been shielded to some extent from the disruptive forces of the pandemic, likely due to deliberate policy and clinical decisions prioritizing pediatric healthcare needs.Interestingly, our study uncovered a statistically significant difference in the gender distribution of surgical patients during the pandemic, with male pediatric patients being more likely to undergo surgical intervention (68.9% vs. 73.5%). This phenomenon warrants further investigation but may be indicative of a broader trend observed during public health emergencies, where healthcare utilization patterns can shift unexpectedly (16). The reasons behind this gender disparity in surgical interventions during the pandemic could be multifaceted, including potential differences in disease incidence, healthcare-seeking behavior, or even the impact of social restrictions on injury patterns.

### 4.2 Categories of surgery

Furthermore, the expectation that the pandemic would lead to a marked increase in the proportion of emergency surgeries, due to delays in elective surgeries and potential exacerbations of health conditions, did not materialize according to our study findings. This observation aligns with reports from other research, suggesting a universal healthcare response aimed at preserving the integrity of emergency surgical services (17). The maintenance of emergency surgeries at levels comparable to pre-pandemic times, despite the widespread postponement of elective procedures, illustrates a significant achievement in healthcare management during a crisis. It reflects a collective commitment within the healthcare community to ensure that urgent and life-saving surgical interventions remain accessible, highlighting the prioritization of critical care services. This prioritization likely involved strategic planning and resource management to balance the need for emergency services with the constraints posed by the pandemic, such as limited hospital beds, personal protective equipment shortages, and the need to minimize virus transmission risks within healthcare settings.

The pandemic's influence extended beyond patient demographics to the very heart of surgical practices. As elective surgeries were postponed or cancelled, the surgical landscape saw a shift towards more urgent and emergent care. This shift is echoed in the work of (18), who discussed the need for surgical departments to adapt rapidly to changing circumstances, ensuring the safety of patients and healthcare workers while maintaining the quality of care.

### 4.3 Index surgery

For index pediatric surgeries, some observed a decline in case numbers, such as esophageal atresia surgeries halving from 5 to 2, and duodenal & intestinal atresia surgeries reducing from 8 to 4. Conversely, others like biliary atresia (Kasai's operation) doubled from 3 to 6, and surgeries for liver tumors emerged from none to 3 cases during the pandemic. In the absence of supportive literature discussing these differences, the authors hypothesized that hepatobiliary surgeries are less commonly performed among different hospitals in Taiwan. The pandemic further enhanced the centralization of such cases.

The analysis further reveals a mixed impact on surgeries for congenital anomalies and other critical conditions, with the numbers for congenital diaphragmatic hernia and choledochal cyst surgeries remaining stable, and a slight increase in surgeries for abdominal wall defects. Moreover, the statistical analysis suggested that the observed changes might not be directly attributable to the pandemic's effects but could also be influenced by other factors such as hospital admission policies and patient hesitancy.

### 4.4 Limitations and future scope

This study is not without its limitations. The retrospective nature of the analysis and the focus on a single institution may limit the generalizability of the findings. The limited number of patients both before and during the pandemics further prohibits the applicability of the study results. Additionally, the dynamic nature of the pandemic, with its waves and varying levels of community transmission, could have influenced the patterns of surgical care in ways not fully captured in this study.

Future research should aim to corroborate these findings with multi-institutional data and explore the underlying causes of the observed gender disparity in surgical interventions. Moreover, prospective studies could provide real-time insights into how ongoing and future public health emergencies affect pediatric surgical practices. Such studies would be invaluable in informing policy decisions and preparedness plans for pediatric healthcare services.

In conclusion, our study contributes to the understanding of the COVID-19 pandemic's impact on pediatric surgical practice in Taiwan. While the age distribution of surgical patients remained stable, the gender distribution varied significantly, and the overall provision of emergency surgical care was resilient. These insights underscore the importance of adaptable healthcare strategies to ensure the continuity and quality of pediatric surgical care during unprecedented times.

## Data Availability

The raw data supporting the conclusions of this article will be made available by the authors, without undue reservation.

## References

[1] Weber DJ Babcock H Hayden MK Wright SB Murthy AR Guzman-Cottrill J Universal pandemic precautions-an idea ripe for the times. Infect Control Hosp Epidemiol. (2020) 41(11):1321–2. 10.1017/ice.2020.313 32616090 PMC7369343

[2] Shiu C Chen WT Hung CC Huang EP Lee TS. COVID-19 stigma associates with burnout among healthcare providers: evidence from Taiwanese physicians and nurses. J Formos Med Assoc. (2022) 121(7):1384–91. 10.1016/j.jfma.2021.07.002 34654583 PMC8501227

[3] Al-Balas M Al-Balas HI Al-Balas H. Surgery during the COVID-19 pandemic: a comprehensive overview and perioperative care. Am J Surg. (2020) 219(6):903–6. 10.1016/j.amjsurg.2020.04.018 32334800 PMC7166034

[4] Babidge WJ Tivey DR Kovoor JG Weidenbach K Collinson TG Hewett PJ Surgery triage during the COVID-19 pandemic. ANZ J Surg. (2020) 90(9):1558–65. 10.1111/ans.16126 32687241 PMC7404945

[5] Cobianchi L Pugliese L Peloso A Dal Mas F Angelos P. To a new normal: surgery and COVID-19 during the transition phase. Ann Surg. (2020) 272(1):e49–51. 10.1097/SLA.0000000000003996 32675493 PMC7268821

[6] Cohen SL Liu G Abrao M Smart N Heniford T. Perspectives on surgery in the time of COVID-19: safety first. J Minim Invasive Gynecol. (2020) 27(7):792–3. 10.1016/j.jmig.2020.04.008 32251839 PMC7129781

[7] Collaborative CO. Elective surgery cancellations due to the COVID-19 pandemic: global predictive modelling to inform surgical recovery plans. Br J Surg. (2020) 107(11):1440–9. 10.1002/bjs.11746 32395848 PMC7272903

[8] Cheng HY Jian SW Liu DP Ng TC Huang WT Lin HH Contact tracing assessment of COVID-19 transmission dynamics in Taiwan and risk at different exposure periods before and after symptom onset. JAMA Intern Med. (2020) 180(9):1156–63. 10.1001/jamainternmed.2020.2020 32356867 PMC7195694

[9] Lin C Braund WE Auerbach J Chou JH Teng JH Tu P Policy decisions and use of information technology to fight COVID-19, Taiwan. Emerg Infect Dis. (2020) 26(7):1506–12. 10.3201/eid2607.200574 32228808 PMC7323533

[10] Romero-Velez G Pereira X Ramos-Santillan V Camacho DR. Surgical outcomes during the first year of the COVID-19 pandemic. Surg Laparosc Endosc Percutan Tech. (2022) 32(5):517–8. 10.1097/SLE.0000000000001001 35960699 PMC9524518

[11] Aguilar JS. Addressing health concerns interrupted by the COVID-19 pandemic. Acta Med Philipp. (2022) 56(6):5–6. 10.47895/amp.v56i6.5635

[12] Dedeilia A Esagian SM Ziogas IA Giannis D Katsaros I Tsoulfas G. Pediatric surgery during the COVID-19 pandemic. World J Clin Pediatr. (2020) 9(1):7–16. 10.5409/wjcp.v9.i1.7 33014718 PMC7515751

[13] Ramachandra C Sugoor P Karjol U Arjunan R Altaf S Halkud R Outcomes of cancer surgery during the COVID-19 pandemic: preparedness to practising continuous cancer care. Indian J Surg Oncol. (2023) 14(2):440–4. 10.1007/s13193-020-01250-z 33100778 PMC7569097

[14] Manja V Wiedeman J Hoch JS Farmer DL. The opportunity cost of changing clinical practice in anticipation of a surge of COVID-19 patients–A convergent mixed methods study protocol. Eur J Person Centered Healthc. (2020) 8(3):301–7. 10.5750/ejpch.v8i3.1793

[15] Rebecchi F Arolfo S Ugliono E Morino M Asti E Bonavina L Impact of COVID-19 outbreak on esophageal cancer surgery in Northern Italy: lessons learned from a multicentric snapshot. Dis Esophagus. (2021) 34(6):doaa124. 10.1093/dote/doaa124 33245104 PMC7717178

[16] Hung KK Walline JH Chan EYY Huang Z Lo ESK Yeoh EK Health service utilization in Hong Kong during the COVID-19 pandemic–a cross-sectional public survey. Int J Health Policy Manag. (2022) 11(4):508–13. 10.34172/ijhpm.2020.248 33105965 PMC9309937

[17] O'Rielly C Ng-Kamstra J Kania-Richmond A Dort J White J Robert J Surgery and COVID-19: a rapid scoping review of the impact of the first wave of COVID-19 on surgical services. BMJ Open. (2021) 11(6):e043966. 10.1136/bmjopen-2020-043966 PMC8210688 34130956

[18] Barry TWL Jeanette TSLJ Ashokka B Lopez KG Thambiah J Kumar N. In the extraordinary times of coronavirus disease 2019: clinical strategies for performing spinal surgery. Asian Spine J. (2020) 14(5):721–8. 10.31616/asj.2020.0146 32872763 PMC7595824

